# Eye movements and ocular biomarkers in mild traumatic brain injury: from oculomotor pathophysiology to diagnosis and prognosis

**DOI:** 10.1007/s00415-026-14035-1

**Published:** 2026-08-05

**Authors:** Ioannis Mavroudis, Oindrila Das, Alin Ciobica, Dimitrios Kazis

**Affiliations:** 1 Third Department of Neurology, Aristotle University of Thessaloniki, Thessaloniki, Greece; 2 Department of Neurology, Leeds Teaching Hospitals NHS Trust, Leeds, UK; 3 Department of Neuroscience, University of Leeds, Leeds, UK; 4 Independent Researcher, New Delhi, India; 5 Department of Biology, Faculty of Biology, Alexandru Ioan Cuza University of Iasi, Carol I Avenue 20Th A, 700505 Iasi, Romania; 6 “loan Haulica” Institute, Apollonia University, Pacurari Street 11, 700511 Iasi, Romania; 7 Academy of Romanian Scientists, 3 Ilfov, 050044 Bucharest, Romania

**Keywords:** Mild traumatic brain injury, Concussion, Eye tracking, Saccades, Ocular biomarker, Prognosis

## Abstract

Mild traumatic brain injury (mTBI) is among the most common neurological presentations worldwide, yet its diagnosis remains largely clinical and its prognosis difficult to predict. Structural imaging is typically normal and self-reported symptom scales are insensitive and confounded by effort and comorbidity. Because the generation and control of eye movements recruit an unusually large and anatomically distributed proportion of the brain—from brainstem and cerebellar circuits to parietal, frontal and prefrontal cortices linked by long white-matter tracts—ocular-motor behaviour is sensitive to the diffuse axonal and neurometabolic disturbance that characterises mTBI. This has motivated intense interest in eye movements as objective, quantifiable and increasingly portable biomarkers of injury and recovery. This review examines the anatomical susceptibility, measurement and current evidence base for the principal ocular domains affected in mTBI: saccades, smooth pursuit, fixation, vergence, the vestibulo-ocular reflex, the pupillary light reflex and accommodation. Across domains the literature converges on a recurring pattern in which cognitively demanding tasks reveal deficits that simple reflexive paradigms miss, supporting the view that ocular-motor dysfunction in mTBI is, in large part, a readout of distributed cognitive control rather than focal damage to ocular-motor machinery. We appraise diagnostic accuracy and the relationship between ocular metrics, symptom burden, white-matter integrity and recovery, and we critically address the methodological heterogeneity that currently prevents any single ocular metric from achieving validated-biomarker status. We argue that the most promising path is a standardised, multi-domain composite, validated longitudinally against converging neuroimaging and clinical endpoints, and we set out the methodological agenda required to bring quantitative eye-tracking into routine clinical practice.

## Introduction

Mild traumatic brain injury (mTBI) is defined by the World Health Organization Collaborating Centre Task Force as a neuro-behavioural syndrome produced by external mechanical forces transmitted to the brain in the absence of a penetrating injury [18]. Although mTBI and concussion are often used interchangeably, as they are in the present review, the terms are not perfect parallels of each other. Concussion is the clinical presentation most often associated with sports while mTBI refers to the wider biomechanical damage cascades in the brain, as defined above. ‘Post concussion syndrome’ (PCS) refers to the set of persistent symptoms an individual experiences beyond a certain recovery timeline, rather than a distinct injury type [70]. To ensure consistency, the term mTBI is held throughout the review, except where research has specifically cited concussion or post-concussion syndrome.

mBI is one of the most frequent neurological events: at least six per thousand people are affected each year, an estimated fifty million cases occur globally per annum, and roughly half the world's population is projected to sustain some form of head injury in their lifetime [59, 71]. mTBI accounts for approximately 90% of all head trauma presentations, with falls, road-traffic collisions and sport- and work-related impacts the leading mechanisms [90]. Although the label “mild” implies benignity, a substantial minority of patients develop persistent post-concussive symptoms, and emerging evidence links repetitive and even sub-concussive impacts to chronic neurocognitive impairment, chronic traumatic encephalopathy and elevated psychiatric and suicide risk [72, 96].

The central clinical problem is that mTBI has no universally accepted diagnostic criterion and no rapidly available objective biomarker to confirm injury or signal recovery [18, 70]. Diagnosis rests on clinical history and symptom assessment, and up to half of patients sustaining an mTBI may receive an inaccurate diagnosis in the emergency department because memory lapses, stress reactions and secondary injury confound assessment [86, 87]. Computed tomography and conventional magnetic resonance imaging are typically normal, and the symptom scales in routine use are limited by reliance on self-report and on clinician experience, missing subtle deficits and underdetecting injury in athletes and others motivated to return to play, work or duty [3, 75]. The consequences of undetected injury—premature return to risk before physiological recovery is complete, and exposure to repeat injury during a window of heightened cerebral vulnerability—make the search for an objective, accessible marker a priority [31].

Eye movements are an attractive candidate. The acquisition and stabilisation of the high-resolution foveal image require precise, rapid coordination of voluntary and involuntary ocular-motor systems, and the neural control of gaze recruits a remarkably large proportion of the brain—retinotectal and thalamocortical relays, primary and extrastriate visual cortex, the middle temporal and medial superior temporal areas, the lateral intraparietal area, the frontal and supplementary eye fields, the dorsolateral prefrontal cortex, the basal ganglia, cerebellum and an extensive brainstem network—interconnected by long white-matter tracts that are themselves vulnerable to the shearing forces of trauma [6, 97, 102]. Because of this distributed substrate, distinct eye movements and their kinematic metrics can be mapped, at least approximately, onto particular regions and functions, so that ocular-motor testing offers a window onto both focal and diffuse dysfunction [11]. The maturation of small, high-speed, non-invasive infrared eye-tracking—and more recently of portable and virtual-reality-integrated systems—has made quantitative measurement feasible outside specialist laboratories, in clinics, on sidelines and at the bedside [97, 95].

This review integrates the evidence across the full range of ocular domains implicated in mTBI and frames it around two translational questions: can ocular metrics serve as diagnostic biomarkers, and can they inform prognosis and recovery? We first summarise the anatomical and neurometabolic basis of ocular-motor vulnerability and the principles and pitfalls of measurement. We then review, domain by domain, the evidence for saccadic, smooth-pursuit, fixational, vergence, vestibulo-ocular, pupillary and accommodative dysfunction, appraising diagnostic accuracy, symptom and imaging correlates, and recovery trajectories. We close with a critical synthesis of the methodological barriers to clinical translation and a roadmap toward a standardised, multi-domain ocular biomarker.

### Scope and methods of the review

This is a narrative review. We searched MEDLINE/PubMed, Scopus and Google Scholar from inception to 2026 using combinations of the terms “mild traumatic brain injury”, “concussion”, “eye tracking”, “oculomotor”, “saccade”, “smooth pursuit”, “vergence”, “vestibulo-ocular reflex”, “pupillometry”, “accommodation”, “biomarker” and “prognosis”, supplemented by hand-searching the reference lists of retrieved articles and relevant systematic reviews. Priority was given to controlled human studies using quantitative ocular measurement, to recent evidence, and to work bearing on diagnostic or prognostic application; animal studies, non-English articles and conference abstracts were not systematically included. The synthesis is interpretive rather than exhaustive, organised to develop the review's central argument regarding cognitive load and distributed control, and should not be read as a systematic review or meta-analysis.

## Why eye movements are vulnerable in mTBI

### Distributed anatomy of ocular-motor control

Visual signals leaving the retina are relayed through the superior colliculus and the lateral geniculate, pulvinar and mediodorsal thalamic nuclei before reaching cortex, where basic visual processing in V1/V2 and V4, the middle temporal area and inferotemporal cortex is integrated with top-down control from the prefrontal cortex, frontal eye field, supplementary eye field and lateral intraparietal area; the striatum, substantia nigra pars reticulata and brainstem generate and gate the movements themselves [11, 94]. Functional imaging shows that saccades, smooth pursuit and optokinetic nystagmus activate largely overlapping cortical and subcortical structures, which is one reason individual ocular-motor tests carry diagnostic value across the syndrome rather than probing isolated pathways [25, 53] Critically, the cortical nodes governing gaze communicate through white-matter tracts — the cingulum and inferior fronto-occipital fasciculus rostrally, and the medial longitudinal fasciculus, medial lemniscus, central tegmental tract and cerebellar peduncles in the brainstem — and these long, oriented fibre bundles are precisely the structures most susceptible to the diffuse axonal injury produced by rotational acceleration [71] Table 1.

**Table 1:** Ocular-motor and ocular domains in mTBI: neural substrate, characteristic abnormalities and biomarker maturity

Domain	Principal neural substrate	Characteristic mTBI findings	Biomarker maturity
Saccades	Superior colliculus, FEF/SEF, DLPFC, parietal cortex, corpus callosum, brainstem	Prolonged latency, reduced self-paced number, anti-/memory-guided errors; reflexive measures often normal	Moderate; best on complex tasks, but poor individual reliability
Smooth pursuit	MT/MST, FEF (frontal pursuit area), pons, cerebellum	Increased lag, reduced gain/accuracy, higher positional error; amplified by cognitive load	Moderate; consistent group effects, calibration-sensitive
Fixation/OKN	Saccadic/pursuit network; brainstem, vestibular relays	Higher fixation velocity and error; reduced OKN gain and symmetry	Low; understudied, instrument-limited
Vestibulo-ocular reflex	Semicircular canals/otoliths, cerebellum, vestibular & oculomotor nuclei	Often normal on quantitative vHIT despite symptom provocation; reduced gain only with controlled rotation; emerging oVEMP changes	Low–moderate; symptom provocation unreliable
Vergence/NPC	Supraoculomotor area, FEF/SEF, superior colliculus, pretectum, cerebellum	Convergence insufficiency (~ 37% of TBI), receded NPC (often doubled), high symptom burden	Moderate–high; clinically tractable, prognostic
Pupillary light reflex	Pretectal olivary & Edinger–Westphal nuclei; sympathetic chain	Slower constriction/dilation velocity, prolonged recovery; sensitive to subconcussive impact	Moderate; objective & portable, confounder-laden
Accommodation	LGN–occipital afferent, Edinger–Westphal efferent, supraoculomotor control	Accommodative insufficiency (~ 43%), slowed/variable response, fatigue; pseudomyopia (rare)	Low; subjective measures, selection bias

*DLPFC* dorsolateral prefrontal cortex, *FEF* frontal eye field, *LGN* lateral geniculate nucleus, *MST* medial superior temporal area, *MT* middle temporal area, *NPC* near-point of convergence, *OKN* optokinetic nystagmus, *oVEMP* ocular vestibular-evoked myogenic potential, *SEF* supplementary eye field, *vHIT* video head-impulse test

### The neurometabolic cascade and diffuse axonal injury

The pathophysiology of concussion is now understood as a neurometabolic cascade rather than a structural lesion. Mechanical deformation triggers indiscriminate glutamate release and ionic flux, an abrupt increase in energy demand met by a period of relative metabolic crisis, mitochondrial dysfunction, altered neurotransmission and cytoskeletal damage to axons [31]. These perturbations can be linked directly to the clinical phenomenology of concussion—migrainous features, transient cognitive impairment and a window of vulnerability to repeat injury—and, with repetition, to mechanisms of chronic dysfunction and neurodegeneration [31]. Because ocular-motor performance depends on the temporal precision of widely separated nodes and on the integrity of the tracts connecting them, even subtle, diffuse and potentially reversible disturbances of this kind can degrade the latency, accuracy and gain of eye movements before they are detectable on structural imaging or naked-eye examination. This provides the mechanistic rationale for treating quantitative ocular metrics as sensitive, if non-specific, indices of network dysfunction Table 2.

**Table 2:** The cognitive-load principle: representative evidence that demanding tasks unmask deficits absent in reflexive testing

*Manipulation*	*Effect on ocular performance*	*Interpretation*
Anti-/memory-guided saccades vs. reflexive	Latency, directional & positional errors increased; reflexive measures preserved	Engages prefrontal/parietal control and connecting white matter
Concurrent word-recall during pursuit	Controls improve synchronisation; mTBI deteriorates	Limited attentional reserve in mTBI
Working-memory (n-back) load during pursuit	Radial-variability diagnostic performance 58% → 79%	Load reveals otherwise subclinical dysfunction
Dual-task (saccade + button press)	Saccade velocity reduced where single-task normal	Compensation fails under divided attention
Natural reading	Reduced saccade amplitude, more fixations/regressions, slower reading	Integrates ocular-motor with cognitive/memory demand

A consistent theme, developed throughout this review, follows from this anatomy: tasks that load cognition and attention—anti-saccades, memory-guided saccades, predictive tracking, pursuit during a concurrent working-memory task, and natural reading—recruit prefrontal and parietal control systems and the white matter connecting them, and it is on these tasks that mTBI-related deficits emerge most reliably, while simple reflexive paradigms are frequently normal [41, 110]. This pattern supports the interpretation that ocular-motor dysfunction in mTBI is, to a substantial degree, a readout of distributed cognitive control rather than damage confined to the ocular-motor apparatus, and it has direct implications for biomarker design.

## Measuring eye movements: instruments, metrics and methodological standards

### Instrumentation and sampling

Eye movements have been recorded since the eighteenth century, progressing from photographic methods through invasive scleral search coils to the small infrared video-oculography systems that dominate current practice [97]. While no agreed gold-standard instruments exist among portable, clinically deployable instruments, the dual scleral search coil offers excellent spatial and temporal resolution, thus becoming a standard point of reference for other methods [91]. It is important to note however that this technique is invasive and not suitable for individuals with acute injuries or within the paediatric population***.*** Structured reviews of the mTBJ literature instead document an array of devices—such as desk- and computer-mounted infrared trackers, rotary-chair video-nystagmography systems, tethered head-mounted units and fully mobile glasses—with sampling frequencies ranging from 60 to 1000 Hz [97]. Sampling rate is not a trivial specification: accurate saccade detection requires at least 50 Hz, and accurate measurement of saccade duration requires of the order of 200 Hz, so that the many studies sampling at 60 Hz can detect that a saccade occurred, but cannot reliably characterise its kinematics, risking the loss of precisely the subtle deficits of interest [5, 58]. Despite this, a structured review found that none of the studies surveyed reported the validity or reliability of their instrumentation, and few reported manufacturer accuracy specifications [97].

### Outcome metrics and their definitions

The kinematic vocabulary is shared across domains: latency (reaction time from stimulus to movement onset), velocity, acceleration, amplitude, gain (the ratio of eye to target movement), duration, frequency and positional or directional error [97]. The difficulty is that thresholds and definitions vary widely between studies. Velocity thresholds for saccade detection in the mTBI literature span 20–240 degrees per second, with higher thresholds used for dynamic walking tasks to exclude vestibulo-ocular contributions; smooth-pursuit paradigms differ in target frequency, waveform, amplitude and visual stimulus; and many studies do not report any detection criteria at all [97]. The choice of threshold materially affects which data are classified as saccade versus fixation and, therefore, what is found, and it helps explain why ostensibly similar paradigms yield discrepant results—for example, smooth-pursuit deficits absent at 0.4 Hz, but present at higher target frequencies [97, 48].

### Confounders: cognition, vision, age, mood and medication

Eye movements are underpinned by cognitive processes from the earliest stage of visual processing onward, yet in one structured review only two of twenty-two studies assessed cognition and only one reported visual-function scores, even though impaired visual acuity or contrast sensitivity independently degrade ocular-motor performance [97, 98]. Refractive correction is a particular concern: spectacles and contact lenses refract the infrared light used to locate the pupil, introducing tracking error and data loss, but the reviewed studies generally did not report whether participants wore correction during testing [97]. Age, depression and centrally acting medication including opioids all influence ocular-motor metrics, and stress and fatigue — endemic to the emergency and sideline settings where mTBI is assessed — alter blink behaviour and gaze control and may even reshape large-scale network connectivity through cortisol-mediated effects [97, 114].

As ocular-motor performance is influenced by normal ageing processes [77] and one’s cognitive reserve raw metrics alone are insufficient in reflecting the actual effect of the injury, often conflating them with pre-existing individual differences. Translating results into standardised Z-scores or using age- and education-adjusted normative references, as done in cognitive testing would offer a more meaningful basis of interpretation at an individual level. However despite its apparent potential, it has not yet been adopted in mTBI literature. Comprehensive reporting and control of these variables is, therefore, not optional, but a prerequisite for interpretable ocular-motor data in mTBI.

## Saccades

### Substrate and clinical measurement

Saccades are rapid conjugate movements that redirect the fovea between points of interest. Although they have an automatic, reflexive component mediated by the superior colliculus and subcortical structures, they are strongly modulated by cognition and attention through the parietal cortex, frontal and supplementary eye fields and dorsolateral prefrontal cortex, particularly during anti-saccades, in which gaze must be directed away from a suddenly appearing stimulus, and memory-guided saccades, made to a remembered location [79, 15]. The corpus callosum and superior colliculus, both central to saccade generation, are demonstrably at risk of diffuse axonal injury in mTBI, providing an anatomical basis for vulnerability [105]. At the bedside, only gross abnormality is detectable: the examiner observes saccades between two targets roughly 30 degrees apart and may note dysmetria with corrective saccades. Office tools, such as the Vestibular/Ocular Motor Screening (VOMS) and the King–Devick and Developmental Eye Movement (DEM), reading tests add symptom provocation or timed performance, but correlate poorly with quantitative eye-tracking and lack objectivity and specificity [8, 29, 78].

### Reflexive (pro-)saccades

Findings on simple reflexive saccades are mixed, consistent with the view that basic paradigms are relatively insensitive. Some groups report prolonged latency and reduced horizontal and vertical accuracy acutely—for example in 28 sport-related concussions tested within two weeks against 87 controls, the only cohort to assess test–retest reliability, which was generally poor for accuracy and pursuit, but acceptable for latency [20]. Others find no overall accuracy difference, attributed to effective corrective saccades, or report latency increases without velocity change [23, 24]. A novel saccade amplitude-to-velocity ratio discriminated mTBI with sensitivities of 0.77 (horizontal) and 0.64 (vertical), although the platform sampled at only 120 Hz with undisclosed analysis [39]. Recurrent weaknesses across these studies—low sampling rates, undescribed calibration and analysis, and proprietary platforms—limit replication Table 3.

**Table 3:** Methodological recommendations for studies of eye movements as biomarkers in mTBI

Domain	Recommendation
Cohort	State explicit mTBI diagnostic criteria; report demographics, recruitment source and comorbidity; stratify by injury stage (acute/subacute/chronic/PCS) rather than pooling
Instrumentation	Report sampling frequency (≥ 200 Hz for saccade kinematics), monocular/binocular recording, calibration procedure and accuracy/precision; report device validity and reliability
Outcomes	Define every eye-movement outcome and detection threshold; use standardised paradigms (e.g. internationally standardised anti-saccade protocol); apply data-reduction to limit multiple comparisons
Confounders	Measure and control cognition, visual acuity/contrast sensitivity, refractive correction, age, mood, medication, fatigue and stress
Design	Use adequately powered samples; include healthy controls and/or pre-injury baselines; perform serial assessment across the acute–subacute transition; report individual-level reliability
Translation	Validate composites against converging neuroimaging and clinical endpoints; benchmark portable/VR and automated pipelines against established methods

*PCS* post-concussion syndrome, *VR* virtual reality

### Complex saccades: anti-saccades, memory-guided and self-paced tasks

Cognitively demanding saccades are more revealing. Reduced numbers of self-paced saccades over a fixed interval have been reported repeatedly; in one persistently symptomatic cohort correlating with disruption of the left uncinate fasciculus and cingulum on diffusion MRI [102]. In acutely concussed athletes, anti-saccade and memory-guided tasks showed prolonged latencies and greater directional and positional errors with larger gain, while reflexive measures were unremarkable, and these deficits were accompanied by widespread fMRI hyperactivation interpreted as compensatory recruitment; partial improvement was seen by 30 days, but performance remained below that of controls [45, 46]. A large emergency-room study of 100 acute mTBIs against 200 controls found impaired pro-saccade error rate and predictive-saccade performance, and a test battery incorporating these measures achieved 89% sensitivity and 95% specificity, with predictive saccades and pro-saccade error rate also tracking recovery at one and two weeks [10, 38]. Increasing cognitive load unmasks deficits otherwise absent: combining saccades with a concurrent button-press reduced saccade velocity in high-school athletes whose reflexive measures were normal [48].

Anti-saccade abnormality, principally in latency and error rate, has been linked to white-matter disruption: increased anti-saccade latency correlated with mean diffusivity of the corpus callosum splenium acutely and the corticospinal tract in persistently symptomatic patients [105]. Paediatric findings are more complex—once children with multiple prior concussions were excluded, acutely concussed children paradoxically made fewer anti-saccade errors, hypothesised to reflect reduced target salience easing reflex inhibition—underscoring the influence of development and prior injury [84]. An artificial-neural-network classifier trained on anti-saccade parameters distinguished mTBI and post-concussion syndrome from controls with 67% and 71% accuracy, but could not separate the two patient groups, implying persistence of anti-saccade abnormality into the chronic phase; the model has not been validated in larger samples [57].

### Dynamic and naturalistic saccade testing

Constrained seated paradigms maximise experimental control, but lack functional validity, because real-world eye movements are goal-directed and embedded in concurrent motor, cognitive and visual demands [97]. Validated velocity-based algorithms now permit saccade quantification during walking and turning in mTBI, and balance-board and virtual-soccer paradigms have revealed increased saccade amplitude and velocity with reduced pursuit velocity, interpreted as a need for more catch-up saccades to compensate for poorly integrated spatial and motion signals [80, 99]. Intriguingly, the direction of the saccade–velocity effect may be task-dependent—reduced in seated paradigms, but increased in dynamic tasks—although the small number of dynamic studies and their methodological variation preclude firm conclusions [97].

### Saccades and recovery

Longitudinal data, though scarce, indicate that complex-task saccadic deficits can outlast symptoms and follow a structured recovery. In a 12-month closed-head-injury cohort, acutely prolonged anti-saccade and memory-guided latencies and directional errors normalised by six months, while absolute position error (accuracy) remained impaired at six months and recovered only by twelve; ocular-motor function at one week predicted overall recovery more sensitively than neuropsychological testing [35, 36]. Persistent post-concussion cohorts show enduring abnormality of anti-saccades, self-paced and memory-guided saccades and pursuit beyond three months, with reflexive measures preserved—again implicating cognitive rather than primary ocular-motor mechanisms [37].

In summary, saccadic dysfunction in mTBI is most reliably elicited by cognitively demanding tasks, with latency the most frequently reported abnormality, followed by gain and positional error; reflexive gain and velocity are poorly sensitive. The major obstacles to biomarker status are heterogeneous sampling rates and protocols and the poor test–retest reliability of complex tasks even in healthy participants, which limits their discriminative value at the individual level and argues for the standardised protocols discussed below [6, 13].

## Smooth pursuit

### Substrate and measurement

Smooth pursuit maintains the image of a moving target on the fovea, integrating motion signals relayed from the middle temporal to the medial superior temporal area and frontal eye field, with the frontal pursuit area encoding motion vectors and brainstem (pontine) and cerebellar circuits matching eye velocity and direction to the stimulus; catch-up and back-up saccades correct residual error [12]. Its dependence on communication between widely separated grey-matter nodes makes pursuit, such as complex saccades, susceptible to diffuse white-matter injury. Clinically it is assessed by having the patient follow a target moving smoothly across the visual field, with the examiner judging saccadic intrusion; the VOMS records only symptom provocation, not pursuit quality [12, 78].

### Pursuit dysfunction across the injury spectrum

Most studies report impaired pursuit in mTBI, characterised by slower tracking velocity, increased lag and higher mean positional error, in both acute and persistently symptomatic patients [19, 61]. Predictive tracking is particularly affected. In 13 acute mTBIs compared with 127 normal and 43 sleep-deprived participants, mTBI showed increased positional-error variability, reduced pursuit radius and reduced velocity; importantly, mTBI was distinguishable from sleep deprivation, with the variability attributed to impaired predictive timing rather than gaze-stability degradation alone [62]. Target-blanking paradigms, which transiently remove the target to isolate cortical predictive control, show shorter time to first saccade, greater intra-individual variability and greater tracking error before and during blanking in mTBI [101].

The cognitive-load principle is especially clear for pursuit. Under a concurrent word-recall task, controls improved their circular-pursuit synchronization—recruiting additional attention—whereas mTBI patients deteriorated [21]. Working-memory load raised the group-wise diagnostic performance of radial pursuit variability from 58 to 79% in patients with protracted recovery, and a repeated attention-demanding task increased pursuit variability selectively in mTBI, indicating task-related fatigability [63, 100]. A portable figure-of-eight pursuit device deployed in hundreds of adolescent athletes used post-test coordinate transformation to dispense with pre-test calibration, an approach that reduces acquisition time and may improve accuracy in sideline settings [49].

Reading and ramp paradigms add detail. In persistently symptomatic military personnel, circular pursuit showed increased intersaccadic intervals, horizontal ramp testing revealed lower gains and longer fixations, and these eye-tracking differences were obtained at 500 Hz in a large, well-characterised sample [112]. Across studies, however, pursuit findings are not universal: where no difference is found, milder injury, greater recovery and larger inter-individual variability are the usual explanations, and several null studies were limited by low sampling rates and unreported calibration — a decisive variable, since a poorly calibrated participant will appear inaccurate irrespective of pathology [64, 65].

### Pursuit, symptoms and imaging in persistent post-concussion syndrome

Pursuit has been studied extensively in post-concussion syndrome, where impairment correlates with symptom severity and, in several cohorts, with white-matter integrity. Higher pursuit error has been linked to attention and working-memory deficits and to reduced integrity of the anterior corona radiata, superior cerebellar peduncle, uncinate fasciculus, forceps major and corpus callosum genu on diffusion MRI [61]. Anticipatory pursuit deficits in chronic mTBI have been associated with suppressed parietal beta-band activity on magnetoencephalography, classifying mTBI with 92% sensitivity in one model, though without reported reliability or replication [26]. A two-centre case–control study found no difference between groups in pursuit error—attributed to injury heterogeneity and differing apparatus—yet still detected white-matter abnormality in the anterior internal capsule and superior longitudinal fasciculus with concordant BOLD changes, illustrating how methodological heterogeneity can obscure a real signal [7].

Overall, smooth-pursuit impairment—increased lag and decreased accuracy, amplified by cognitive load—is among the more consistent ocular findings in mTBI and correlates with symptom burden and injury severity. As with saccades, the dominant influence of cognition both strengthens its appeal as an index of network dysfunction and complicates its specificity; calibration reporting and protocol standardisation are the principal unmet needs.

## Fixation and optokinetic nystagmus

Fixation and nystagmus are comparatively understudied. Fixation is intimately related to saccades—fixations are the pauses between saccades, which may explain why dedicated fixation outcomes are often subsumed within saccade and pursuit analyses [56].

Although smooth pursuit occurs when a patient fixates on the target, pursuit and fixation are not the same motor behaviours clinically or neurophysiologically. Patients with congenital nystagmus can fixate on a stationary task, but display jerky pursuit movements [50]; vestibular nystagmus on the other hand is suppressed by fixation and expressed during smooth pursuit of a stationary target [54]. Moreover, at the cellular level, the frontal eye field fixation cells show a reduction in their discharge rate during smooth pursuit, and microstimulation in this area suppresses smooth pursuit mechanisms. Hence, while fixation and pursuit share some commonalities, these eye movements are regulated by distinct neural circuits [43, 44].

When examined, mTBI is associated with greater fixation accuracy and initial-fixation error, higher absolute fixation velocity and longer fixation duration, and during reading with more and shorter fixations [24, 112].

Optokinetic nystagmus is difficult to capture with infrared trackers because measurement is influenced by head position and high sampling rates are required, so electro-oculography has traditionally been preferred; moreover, the sustained stimuli that evoke nystagmus frequently exacerbate mTBI symptoms, forcing test cessation and reducing usable cohort sizes [83, 34]. Nonetheless, reduced optokinetic slow-phase gain, gain asymmetry and increased gain variability have been reported in mTBI and shown to track recovery, suggesting that with appropriate instrumentation this domain merits further study [48, 10].

## Vestibulo-ocular reflex

### Substrate and measurement

The vestibulo-ocular reflex (VOR) stabilises the retinal image during head movement through peripheral vestibular input from the semicircular canals and otolith organs, central integration via the cerebellum, vestibular and oculomotor nuclei, thalamus and reticular formation, and motor output to the extraocular muscles [22]. mTBI may disturb the system peripherally—blast pressure waves damaging inner-ear sensory organs—or centrally, through diffuse axonal injury and microhemorrhage in the cerebellum [51, 1]. Clinically the VOR is tested by rapid 20-degree head rotation with fixation on a target; a corrective saccade signals failure. The video head-impulse test (vHIT) quantifies VOR gain and corrective saccades objectively, while caloric and rotational-chair testing probe restricted aspects of the system [77, 28].

### Evidence and the symptom-provocation problem

A central difficulty in this domain is that most studies report only symptom provocation during VOR testing—dizziness or nausea on head rotation—which does not demonstrate VOR dysfunction, since such symptoms may arise from non-vestibular sources; symptom provocation is in fact a poor indicator of vestibular dysfunction [2, 9]. The VOMS, which rates symptom provocation across a battery of gaze tasks, had an originally reported sensitivity of 50% for item scores of two or more and may help identify protracted recovery, albeit with frequent false positives [78, 52]. When the VOR is measured quantitatively, results are largely negative: multiple vHIT studies in adolescents and adults found normal VOR gain despite high symptom scores, supporting the conclusion that symptom provocation is insensitive to true vestibular dysfunction [4, 27, 93]. A notable exception used a computer-controlled rotational head-impulse paradigm in 100 acute mTBIs, finding reduced gain and inter-ocular asymmetry that persisted at two weeks, particularly in symptomatic patients—suggesting that only more controlled, quantitative methods detect genuine VOR abnormality [10, 38].

Ocular vestibular-evoked myogenic potentials (oVEMPs), which probe utricular otolith function, are an emerging and potentially more sensitive measure. Abnormal or absent oVEMP responses were more prevalent in contact-sport athletes with a history of mTBI than in non-contact athletes or controls, attributed to the utricle's vulnerability to repetitive trauma, and prolonged oVEMP latencies were found in military personnel with previous mTBI, more pronounced in those with anxiety, depression and post-traumatic stress [92, 73]. The field is in its infancy; realising oVEMPs or combined vHIT–oVEMP protocols as biomarkers will require standardised methods and larger longitudinal studies. Overall, the VOR illustrates a recurring lesson: symptom-based vestibular assessment is unreliable, and only precise, quantitative measurement with adequate control discriminates injury.

## Vergence and convergence insufficiency

### Substrate and measurement

Vergence comprises the non-conjugate movements that align both eyes on near (convergence) or far (divergence) targets, served by the midbrain supra-oculomotor area, frontal and supplementary eye fields, superior colliculus, pretectum and cerebellum. A proposed mechanism of mTBI-related vergence impairment is a global processing delay in afferent pathways, manifested as increased latency and reduced velocity of both convergence and divergence [103]. Clinically, near-point of convergence (NPC)—the nearest point at which single binocular vision is maintained—and fusional vergence ranges are measured, and convergence insufficiency (CI) is diagnosed when defined criteria of exophoria, a receded NPC and reduced positive fusional vergence are met, following the diagnostic criteria established by the Convergence Insufficiency Treatment Trial [89, 116]

### Prevalence, symptoms and recovery

Vergence dysfunction is among the most clinically tractable ocular sequelae of mTBI. Against a non-presbyopic population prevalence of CI of roughly 2–13%, pooled prevalence in TBI reaches 37.2% (95% CI 24.3–51.1%) in a systematic review and meta-analysis, and NPC distance has been reported, on average, to double in mTBI [76, 103]. CI carries a high symptom burden: in adolescents with recent mTBI, attention, learning and memory were significantly worse in those with CI, and the majority had overlapping vergence, accommodative and ocular-motor disorders [82]. Receded NPC is more prevalent in persistently symptomatic patients and correlates with worse verbal memory, slower visual-motor speed, longer reaction time and greater symptom burden, making it a useful marker of severity and recovery; in contact-sport players, NPC recedes mid-season with subconcussive impact exposure and recovers post-season [81, 115].

Imaging studies link CI to disrupted fractional anisotropy in the anterior thalamic radiation and geniculate optic tracts and to reduced activation of the lateral geniculate, superior colliculi, oculomotor and abducens nuclei and supraoculomotor area, though several are small, unblinded or lacking baseline data [1, 109]. Combining vergence metrics from binocular eye-tracking with NPC status improved classification of mTBI in a paediatric cohort, an example of multi-metric integration moving toward clinical utility [14]. Prognosis is mixed: blast-induced cohorts show persistent CI years after injury, and a substantial fraction of TBI-related CI persists beyond 12 months. Divergence remains comparatively unexplored. Larger longitudinal trials controlling for age, time since injury and severity are needed before vergence indices, alone or combined, can be adopted as biomarkers [55, 60].

## Pupillary light reflex

### Substrate and measurement

The pupillary light reflex (PLR) balances retinal illumination through parasympathetic constriction and sympathetic dilation. Afferent signals pass from retinal ganglion cells to the pretectal olivary nucleus and Edinger–Westphal nuclei, whose efferents drive the iris sphincter, while the sympathetic pathway descends from the hypothalamus through the superior cervical ganglion to the dilator pupillae [66, 88]. Clinical grading on a 0 to 4 + scale is crude; quantitative infrared pupillometry resolves constriction latency and amplitude, constriction and dilation velocities and recovery time, and is well suited to portable deployment [88].

### Evidence across acute, subconcussive and chronic injury

Pupillometry has yielded some of the more reproducible objective findings in the field. In 100 military personnel with acute mTBI against 100 controls, constriction and dilation velocities were slower and 75% recovery time prolonged, with constriction latency, average constriction and dilation velocity and recovery time all impaired between 15 and 45 days post-injury [16]. In adolescent athletes followed across a season, the light reflex was briskly enhanced on the day of injury and then showed reduced constriction and dilation velocities during recovery, while football players sustaining subconcussive impacts showed decreased dilation and constriction velocity and altered resting diameter without a corresponding symptom change—raising the possibility that pupillary metrics index subclinical trauma [85, 47]. Chronic mTBI cohorts show reduced constriction and dilation velocity and amplitude, with the authors attributing slowed dilation to sympathetic and reduced peak velocities to parasympathetic involvement, although these studies are limited by selection bias toward symptomatic patients, inadequate age-matching and confounding by migraine [104, 107].

The principal caveat is the long list of confounders that shape pupillary dynamics — refraction and ocular biometry (high myopes show the slowest velocities), perceptual awareness, attention, mental imagery, pain, anxiety, arousal, opioids, alcohol, migraine history and cognitive load [108, 67]. Monocular pupillometry has been validated as symmetric in mTBI, simplifying acquisition, but meticulous control of these factors is a precondition for establishing diagnostic utility. The PLR is promising precisely because it is objective, rapid and portable, yet remains short of biomarker status pending confounder-controlled, longitudinal validation.

## Accommodation

Accommodation adjusts the optical power of the eye through ciliary-mediated lens thickening and pupillary constriction, governed by an afferent limb through the lateral geniculate to occipital cortex, an efferent limb from the Edinger–Westphal nucleus, and oculomotor control neurons that translate retinal blur into motor command via visual-association cortex and supraoculomotor nuclei [69]. It is measured clinically by amplitude of accommodation and accommodative facility, with accommodative insufficiency defined by an amplitude two or more dioptres below the age norm or facility of six cycles per minute or fewer [30].

Accommodative insufficiency is common after mTBI, especially in paediatric and non-presbyopic cohorts, though prevalence is probably overestimated by small samples, referral bias and mixed injury severity; a meta-analysis of mixed-cause TBI cites 42.8–43% [76, 32]. Objective laboratory study using open-field autorefraction found increased time to accommodate, reduced peak velocity — slowed up to fourfold — variable amplitudes and pronounced accommodative fatigue in mTBI, although confounded by pharmacological factors, selection bias and mixed aetiology [33]. Prognosis is poorly characterised: roughly a third of military personnel show persistent accommodative dysfunction beyond a year, while a Swedish cohort found half persistently impaired at follow-up, in contrast to convergence which recovered [17, 68]. Post-traumatic pseudomyopia (accommodative excess) is largely anecdotal, with some cases attributable to ciliary spasm and ciliochoroidal effusion resolving within weeks and others persisting [42]. Evidence for accommodation is constrained by reliance on subjective clinical measurement and selection bias; its principal clinical value may lie in near-vision metrics as predictors of delayed return to school or work.

## Ocular metrics as diagnostic and prognostic biomarkers

### Diagnostic performance

A biomarker, in the Biomarkers Definitions Working Group sense, is a characteristic objectively measured as an indicator of normal or pathogenic processes or response to therapy, and mTBI requires markers for three distinct tasks: diagnosing acute injury, characterising persistent post-concussion syndrome and confirming recovery [117]. The best diagnostic performance reported comes from multi-domain batteries rather than single metrics. Computer-controlled oculomotor, vestibular and reaction-time testing achieved 89% sensitivity and 95% specificity in acute mTBI, and a magnetoencephalography-coupled pursuit model reached 92% sensitivity, while single-task figures are more modest—anti-saccade neural-network classification at 67–71%, baseline pursuit variability at 58% rising to 79% under working-memory load [10, 57,26,100]. Importantly, the apparently strong performance of some emergency-department applications is not universal: an exploratory study using virtual-reality-integrated eye-tracking in 82 acute patients found only two of 52 parameters (blink rate and eyelid-closure frequency) significantly discriminated mTBI, both with modest area under the curve of about 0.63, and concluded that oculomotor abnormalities may not be robustly detectable within the first 24 h [95]. This negative result is instructive: it reflects both the genuine difficulty of acute-phase detection and the methodological constraints—manual annotation, absence of healthy controls, exclusion of open-head injuries—that beset real-world deployment.

### Timing of detectability

Several lines of evidence suggest that ocular-motor abnormality is more pronounced in the subacute phase, roughly three to fourteen days after injury, than in the first 24 h, when acute stress, fatigue and cortisol-mediated network changes may dominate and obscure injury-specific signals [95, 113]. This has practical implications: the optimal window for ocular biomarker testing may not coincide with emergency presentation, and serial assessment across the acute-to-subacute transition is likely to be more informative than a single early measurement.

### Prognosis and recovery

The prognostic signal is, if anything, stronger than the diagnostic one. Ocular-motor function at one week after injury predicted overall recovery at three and six months more sensitively than neuropsychological assessment, and structured recovery trajectories—earlier normalization of latency and directional error, later normalization of accuracy—have been documented over 6–12 months [35, 36]. Predictive saccades, pro-saccade error rate and optokinetic gain symmetry differentiate mTBI from controls and track recovery over the acute and subacute period [38]. Near-vision measures are particularly promising prognostically: receded NPC correlates with symptom burden and neurocognitive impairment and may forecast delayed return to school or work, and its resolution could in principle inform return-to-play decisions [81]. Across domains, the persistence of complex-task saccadic, pursuit and vergence deficits beyond symptomatic recovery suggests that ocular metrics may index biological recovery that lags clinical recovery—precisely the gap that return-to-risk decisions need to bridge [37].

### Methodological barriers to clinical translation

Despite a large and growing literature, no single ocular metric has achieved validated-biomarker status, and the reasons are predominantly methodological. A structured review of the eye-tracking literature in mTBI identifies a consistent set of deficiencies that recur across every domain surveyed here [97].

#### Instrumentation and signal quality

Sampling frequencies vary from 60 to 1000 Hz, and many studies sample too slowly to characterise the kinematics they report; validity and reliability of instruments are essentially never reported; and calibration, monocular versus binocular recording, infrared versus video methods and artefact control are inconsistently described [97, 5].

#### Outcome definition and analysis

There is no consensus on detection thresholds, eye-movement definitions or processing algorithms; velocity thresholds for saccades range over an order of magnitude; and many studies report large numbers of outcomes without correction, inviting type I error, while small cohorts simultaneously risk type II error and missed small effects [97, 95].

#### Cohort characterisation and confounders

mTBI lacks a universally accepted definition, so diagnostic criteria are frequently unstated; demographic reporting is often incomplete; injury stage (acute, subacute, chronic, post-concussion syndrome) is variably defined and sometimes collapsed into single cohorts, obscuring stage-specific effects; and cognition, visual function, refractive correction, age, mood and medication are seldom measured or controlled [97, 98].

#### Reliability at the individual level

Even a standardised saccade protocol shows excellent reproducibility for basic measures, but poor reproducibility for the complex tasks (anti-saccades, double-step saccades) that are most sensitive to mTBI—a fundamental tension, since the most informative tasks are the least reliable in individuals, and this variability can only increase in disease [13]. Protocols are also long: a 21-min standardised battery may be impractical in symptomatic patients with attentional deficits and symptom provocation [6, 13].

These problems are compounded by selection bias toward concussion-clinic populations, in whom visual symptom prevalence is inflated, and by a paucity of community and subconcussive data, where most injuries occur, but go unstudied [74]. The net effect is a literature rich in promising single-study findings, but poor in replication, standardisation and individual-level validity.

### Toward a clinically useful ocular biomarker

The evidence reviewed here points away from the search for a single decisive metric and toward a standardised, multi-domain composite. Several principles follow from the foregoing analysis.

First, standardisation is the precondition for everything else. The field needs agreed instrument specifications (minimum sampling frequency, mandatory reporting of accuracy, precision and calibration), agreed outcome definitions and processing algorithms, and adoption of internationally recognised paradigms, such as the standardised anti-saccade protocol, so that results can be pooled and replicated [97, 13, 40].

Second, composite metrics that combine domains—for example, vergence indices with smooth-pursuit accuracy and complex-saccade measures—are likely to outperform any single task, because they sample the distributed network more completely and because cognitively loaded paradigms consistently outperform reflexive ones [110]. Data-reduction techniques, such as principal-component or factor analysis, can convert the unwieldy array of correlated outcomes into interpretable domains and reduce multiple-comparison risk [97]. This ‘distributed’ sensitivity has an important implication; rather than being unique to the mechanism of mTBI, it is likely to index broader cognitive and attentional control.

Third, validation must be longitudinal and multimodal, anchoring ocular metrics to converging endpoints—diffusion and functional MRI, magnetoencephalography, blood-based markers and clinical outcomes—and establishing diagnostic and prognostic accuracy at the individual rather than group level, with explicit attention to the optimal testing window across the acute-to-subacute transition [61, 7, 113].

Fourth, confounders must be measured and modelled rather than assumed away: cognition, visual function, refractive correction, age, mood, medication, fatigue and stress should be standard covariates [97, 108].

Finally, translation requires instruments that are reliable, portable, rapid and inexpensive, and that integrate with multimodal assessment, so that quantitative ocular-motor testing can move from the laboratory to the clinic, the sideline and the bedside. Portable and virtual-reality systems, calibration-free coordinate-transformation approaches and automated analysis pipelines are encouraging steps, provided their accuracy is validated against established methods [95, 49].

An important implication that naturally follows the central argument of this review is that if the sensitivity of ocular motor measures demonstrates the distributed networks of cognition and attention that control eye movements, then a composite multi-domain measure should not be considered an mTBI-specific tool. Rather, it can be better understood as a potentially eclectic indicator of the integrity of the brain networks. Considering this angle, its clinical utility may extend well beyond mTBI to a diverse range of neurological and psychiatric conditions; thus, making ocular- motor performance a shared and quantifiable marker of brain function across disorders [106, 111].

## Conclusion

Eye movements are impaired across the spectrum of mild traumatic brain injury—in saccades, smooth pursuit, fixation, vergence, the vestibulo-ocular reflex, the pupillary light reflex and accommodation—reflecting the diffuse, network-level disturbance that characterises the injury and the unusually distributed neural substrate of gaze control. The most consistent and informative deficits emerge on cognitively demanding tasks, supporting the view that ocular-motor dysfunction in mTBI is largely an index of distributed cognitive and attentional control rather than focal ocular-motor damage. Ocular metrics show real promise as objective, quantifiable and increasingly portable markers, with particular strength in prognosis and in detecting biological recovery that outlasts symptoms. Yet the evidence is not yet sufficient to adopt any single quantitative ocular-motor assessment in routine clinical practice. The barriers are methodological—heterogeneity of instruments, protocols, outcome definitions and cohorts, inadequate confounder control, and poor individual-level reliability of the most sensitive tasks—and they are tractable. A standardised, multi-domain, longitudinally validated ocular composite, benchmarked against converging neuroimaging and clinical endpoints and delivered through portable, automated instruments, represents the most credible path from the current array of promising findings to a clinically deployable biomarker for the diagnosis, prognosis and management of mild traumatic brain injury.

It is important to note also that alongside mild traumatic brain injury, the line of reasoning pursued in this review suggests that standardised ocular-motor composites may serve as a quantitative substitute for distributed brain networks and their functions, an avenue with the potential of research across neurological and psychiatric conditions.

## Data Availability

Data sharing is not applicable to this article as no new data were created or analysed.

## Declarations

### Use of large language models

A large language model was used to assist with grammatical review and reference formatting during preparation of this manuscript. All content was reviewed, verified and edited by the author(s), who take full responsibility and accountability for the final text and for the accuracy of all statements and references.
